# See think act scale: Validation of the Dutch version of a measure of relational security in high secure forensic psychiatric care

**DOI:** 10.3389/fpsyt.2022.1020718

**Published:** 2022-10-03

**Authors:** Meike G. de Vries, Robbert-Jan Verkes, Berend H. Bulten

**Affiliations:** ^1^Forensic Psychiatric Center Pompestichting, Division Diagnostics Research and Education, Nijmegen, Netherlands; ^2^Department of Criminal law, Law School, Radboud University Nijmegen, Nijmegen, Netherlands; ^3^Donders Institute for Brain, Cognition and Behavior, Radboud University Medical Centre, Nijmegen, Netherlands; ^4^Faculty of Social Sciences, Radboud University Nijmegen, Nijmegen, Netherlands

**Keywords:** relational security, team reflexivity, ward climate, forensic care, psychometrics

## Abstract

Relational security is considered an essential form of security in forensic psychiatric care. Research on relational security is important, but is hampered by the lack of instruments to assess and monitor this concept in clinical practice. Within this current study the psychometric properties of the Dutch version of the See Think Act (STA) scale, an instrument designed to measure relational security as perceived by forensic staff members within secure settings, was studied. Results show that the internal consistency of the STA total scale was good. However, the internal consistency of the subscales was relatively low compared to other studies using the original English or the Chinese version of the STA scale. The factor structure found in the original English version of the scale was not confirmed within this sample. With regard to the validity of the instrument results were promising, finding relationships with aspects of ward climate and team reflexivity. Further research and development is needed regarding the STA scale, making it more suitable for monitoring and studying this clinically relevant concept in forensic care.

## Introduction

Within (high) secure forensic psychiatric care, three domains of security are used in order to maintain safety throughout the recovery process of patients, namely physical security, procedural security and relational security (1–3). Physical security refers to elements in the environment such as perimeter fences and electronic alarm systems. Procedural security refers to policies and practices such as unit and room searches or drug controls. While these first two forms of security are rather clearly described or even tangible, the third form seems to be harder to define. Relational security has been divided into two aspects, a quantitative and qualitative one (4). Quantitative relational security includes variables such as the staff-to-patient ratio, and the amount of time spent in face-to-face contact. Qualitative relational security in general, relates to maintaining a therapeutic relationship with trust, while managing boundaries so that risk is recognized and managed, implying a need for in depth knowledge about patients (2). There is no consensus on a definition of relational security yet. Hence, there are several definitions showing both variance and overlapping issues. Tighe and Gudjonsson (5) focused in their definition of relational security on the quality of the therapeutic relationship clinicians have with their patients and the way this relationship is used to maintain safety through the recovery process. The Department of Health, (6) (DoH) in the United Kingdom referred to relational security as the knowledge and understanding staff have of a patient and of the environment, and the translation of that information into appropriate responses and care. Hence, using knowledge of patients risks and needs, enables tailored security measures as levels of restriction and supervision can be varied according to the needs of the patient while maintaining the safety of others (7, 8). In an integrative review of the literature on relational security, Fletcher (9) identified therapeutic relationship, ward climate and team dynamics as the tree main themes playing a role in relational security. Based on her findings Fletcher ((9) pg. 73) extends former definitions of relational security: Relational security is “*the detailed clinical knowledge of a patient and the translation of this knowledge into safe management of their care. It is also the organization of the wider ward, including the management of increased acuity and the therapeutic program. Finally, it is the understanding of staff dynamics and the impact this has on effective communication within the team and the translation of clinical knowledge to the delivery of patient care.*”

The Department of Health (6) in the United Kingdom published “See Think Act (STA),” a handbook including a model that could help professionals working in forensic care in evaluating and maintaining relational security. The STA model is based on an analysis of a series of ward incidents in low to medium-secure forensic services in the United Kingdom. It was found that most incidents where related to a breakdown in the interpersonal and risk-management aspects of care, that one could categorize as relational security aspects (5). The purpose of the STA model is to help staff understand what relational security means, it offers structured guidance for clinical teams that encourages relational security by the maintenance of security and vigilance while promoting patient recovery (10). The STA model has four components; (1) a team’s ability to maintain boundaries and deliver therapy, (2) patient mix and inter-patient dynamics, (3) the internal world of the patient and the unit, and (4) connections to the outside world and the impact of visitors. In the STA handbook each component of the model, and its relevance for relational security in clinical practice, is described. At the end of each section of the handbook, statements are presented to prompt reflection among staff members on their practice.

The STA method consists, apart from the handbook of additional tools like a workbook with exercises, a format to map a patient-mix and explorers to help evaluate relational security issues. The handbook and the tools can be considered as a starting point in helping professionals to explore and fulfill their role in relational security. In order to integrate the STA method in daily practice, professionals need training, encouragement, support and robust strategic leadership with an emphasis on reflective practice (11). Organizations need to educate and train their staff, have a structure in place that support ongoing skill development in delivering relational security care, and have clear and effective systems for communication and handover within and between staff teams (12). It has been argued that in secure and forensic mental health settings the humanistic values that underpin nursing can be in conflict with actual practice. The dual role that staff members have in therapy and control, combined with the need for personal safety for professionals, might result in adapting more custodial and restrictive than care related attitudes and practice (13, 14). For instance, distancing yourself as professional from patients has been mentioned as a way to cope with relational difficulties (15). However, in order to enable recovery, relationships and environments that provide hope, empowerment, choices, and opportunities for fulfilling an individual’s potential are required (16). Relational security could support forensic mental health professionals in finding balance in managing safety and risks and patients’ recovery and care (11). However, there is a need for studies into the actual impact of relational security on for instance, risk incidents on the ward, treatment outcomes and patient satisfaction as there are no results yet that underwrite the potential beneficial effects of relational security (7). Within the United Kingdom all forensic facilities are encouraged to work on their relational security using the STA guidelines. In cooperation with the author of the original STA guideline, a Dutch translation was published (17) making the material accessible for professionals working in forensic psychiatric care in Netherlands. In the Dutch translation of the STA handbook the definition of relational security was enriched, with approval of the original author, by adding the importance of self-knowledge of staff. Resulting in the following definition: “… *the knowledge and understanding staff have of a patient, themselves and of the environment, and the translation of that information into appropriate responses and care*.” This adjustment was made after experiencing in clinical practice that reflection on oneself as a professional, is important in working with patients as well as in working in a team. The addition to the definition has also had implications for the use of the STA model, by putting more emphasis on reflective practice, which is in line with recent thinking by both Fletcher (9) and Markham (11). Considering good communication and information sharing, as the corner stone of relational security and recovery-focused care. In forensic facilities, working multi-disciplinary as a team and using expert skills are considered essential for effective risk management and appropriate patient care (18, 19). Markham (11) argues that reflective practice in forensic care is important for staff members and teams to gain insight, learn from daily practice and optimize relational security. The STA guidelines recommend to engage in reflective practice within the multidisciplinary team in order to evaluate and improve relational security and patient care. In general, reflective practice encompasses a process in which teams regularly engage in situated action, reflect on the experience, extract learning’s and plan how to integrate those learning’s into further actions (20–22). Team sessions such as debriefs in which teams discuss, interpreted and learn from recent events are widely used in (mental) healthcare settings and are found to be related team effectiveness when well conducted (23, 24). In the literature the term team reflexivity is used, referring to the extent to which team members collectively reflect upon the their team’s objectives, strategies and processes (25, 26).

Although relational security is considered an essential form of security in forensic psychiatric care, it has received limited research attention in clinical practice. There is a lack of data on the implementation of relational security in inpatient settings (9, 11). The lack of studies concerning relational security could be related to the challenges in defining the concept of relational security and isolating the essential elements. In an attempt to fill this gap, Tighe and Gudjonsson (5) developed a measure of qualitative relational security (See Think Act Scale, STA Scale) as perceived by forensic staff members, based on the content presented in the STA DoH practice guidelines (6). The original English version of the STA Scale has been found to have high levels of internal consistency and moderate to good convergent validity with instruments partly addressing aspects of relational security (5, 7, 27). Tighe and Gudjonsson (5) used the three subscales (therapeutic hold, experienced safety and patient cohesion) of the EssenCES (28, 29), a measure of ward climate, to establish construct validity of the STA Scale. They reported moderate to strong positive correlations between relational security and two subscales of the EssenCES measuring patient cohesion and therapeutic hold, within a sample of 159 nursing staff members working in a forensic low and medium-security service. Arsuffi (7) used the EssenCES total scale and found a moderate positive relationship between relational security and the EssenCES total score within a sample of 58 staff members working on low secure, medium secure and open rehabilitation sites in England. Altogether, there are indications that the STA scale has operationalized the concept of relational security in a way that it can be measured with fair reliability and promising validity, however, more research is needed. The aim of this present study was to examine the psychometric qualities of the recently developed Dutch version of the STA scale. To investigate construct validity the relationship between the scores on the STA scale with scores on established instruments to measure ward climate and team reflexivity was assessed. Although relationships between these concepts need to be studied in clinical practice, we can make some assumptions based on earlier work regarding these concepts. Hence, as relational security is based on “the knowledge and understanding staff have of a patient, themselves and of the environment, and the translation of that information into appropriate responses and care” it could be argued that reflective practice is an important factor for optimizing relational security. Hence, discussing processes and evaluating practice within a team, contributes to knowledge and insight which can be translated into appropriate responses and care. Within this current study it is hypothesized that higher scores on evaluation and learning and discussing processes [two subscales of the Team Reflexivity Scale; (30)] would relate to higher scores on the STA scale.

Good practice on relational security should translate into aspects of ward climate like therapeutic hold. Hence, earlier work has found that ward climate and relational security have been found to be concepts that are moderately related to each other (5, 7). Both concepts are found to be important factors in high secure forensic care, and in both concepts the therapeutic relationship between staff and patients plays an essential role. However, looking at the definitions used for relational security, this concept concerns skills of staff members in preforming their job, translating their knowledge and observations into appropriate responses to maintain and enable both safety and recovery. While ward climate can be considered as a dynamic and multifactorial construct, which describes the social and emotional experience of a unit by its staff or residents (28, 31, 32). Based on the results of the earlier studies mentioned above it was hypothesized that staff’s perceptions of relational security would be positively related to two elements of ward climate, namely therapeutic hold and patient cohesion [as measured with the EssenCES; (28, 29)].

## Materials and methods

### Participants

Data were collected at several wards of the Pompestichting, consisting of a high secure forensic psychiatric institution for male patients and a high security long term forensic psychiatric care (LFPC) facility in Netherlands. The total sample consisted of 99 (61 women) staff members working on the wards in the day to day care. In Netherlands these staff members are often referred to as “sociotherapists.” In general the educational background of sociotherapists is higher education (e.g., Social Work, Nursing). The average age of the respondents was 37 years old (range: 21–65, SD: 12.1). The average work experience in their specific function was 8 years (range 0.25–40, SD: 8.1).

### Measures

#### Relational security

Relational security was measured using the See Think Act (STA) scale (5). The STA scale is a 28-item self-report scale, designed to measure relational security as perceived by forensic staff members within secure settings. The STA scale consists of four subscales: therapeutic risk management; pro-social team culture; boundaries; and patient focus. Responses are made on a 4-point scale, ranging from “just like our team” to “not like our team.” Examples of items representing the different factors are: “We are vigilant about how visits affect the patient before their visit” (therapeutic risk management) “We deal with bullying robustly” (pro-social team culture), “we understand why maintaining a clear boundary with patients is important’ (boundaries),” “Care plans are up to date to reflect how our patients are feeling today” (patient focus). Permission was granted by Tighe, the original author of the STA scale to translate the English version into Dutch. First, the questionnaire was translated from English to Dutch by an academic-scientific translation agency for academia and research, and then back translated by another professional of the agency. The original English version and the translated version where compared and differences discussed by the authors of this current study, an independent researcher and one of the translators, modifications were made, resulting in the Dutch translation of the STA scale used within this study. The Dutch version of the STA-scale, used within this study, can be found in the supplement.

#### Ward climate

Ward climate was measured using the EssenCES (28, 29). The EssenCES is a 17-item questionnaire. Ratings were obtained using a 5-point likert scale ranging from “I do not agree” up to “totally agree.” Examples of items representing the different factors are “The patients care for each other” (patient cohesion); “Really threatening situations can occur here” (experienced safety); on this ward, patients can openly talk to staff about all their problems’ (therapeutic hold).

#### Team reflexivity

Team reflexivity was measured using the Team Reflexivity Scale (30). The scale consists of two subscales: “evaluation and learning” and “discussing processes.” The evaluation and learning scale focuses on the evaluation of finished business and learning from previous actions and adaptations. Discussing processes focuses on thinking about the way things are usually done in the team, reflecting on communication patterns on norms and values within the team. Ratings were obtained using a 5-point Likert scale ranging from “totally disagree” up to “totally agree.” Examples of items representing the two factors are “We work out what we can learn from past experiences” (evaluation and learning); “The methods used by the team to get the job done are often discussed” (discussing processes).

### Procedure

Data collection was part of a larger project within the Pompestichting monitoring multidisciplinary teams during the implementation of a model developed to aid professionals in enhancing relational security [See Think Act; (12, 17)]. During this implementation project multidisciplinary teams, received a 1 day training in relational security each year. During that training the origin, core elements and use of the STA model are explained and practiced. Data collection took place from February 2022 to June 2022, approximately after all teams had received at least 1 or 2 relational security training days. The study was approved by internal review board (Scientific Committee) of the Pompestichting and was conducted in accordance with the Declaration of Helsinki (33). Participation was voluntary, after receiving oral and written information concerning the data collection, the study aims and objectives, participants signed an informed consent form. The study consisted mostly of a paper-and-pen data collection. Participants were granted approximately 20 min of time during a general team meeting to fill out the questionnaire. The questionnaire, consisted of questions concerning age, education, work experience and gender, followed by the measures of ward climate, relational security and team reflexivity. The questionnaires were returned to the investigator in a closed envelope. Only a few teams were asked to fill out an online version of the questionnaire, as they indicated that they did not have time to fill out the questionnaire during an upcoming team meeting. In both versions of the questionnaire (pen and paper, and online) participants were asked at the end, whether they wanted to participate in a second part of the study by filling out one of the scales (the STA scale) again in one or 2 weeks’ time. Participants who were willing to do that, wrote down their email address so that the researcher could send them the second measure. This effort resulted in a subgroup of 19 participants that filled out the STA scale two times to get insight in the test –retest reliability of the scale. After the data collection, the data was anonymously analyzed, to ensure that participants could not be identified based on the data during data analyses and reporting.

### Statistical analyses

Internal consistencies of the (sub)scales are calculated using Cronbach’s alpha. Confirmatory factor analysis (CFA) was used to see whether the original factor structure as suggested by Tighe and Gudjonsson (5) was retained within this study. The robust maximum likelihood (MLR) estimation procedure was used to account for non-independence and non-normality (34). The fit of the model was examined using the Root Mean-Square Error of Approximation (RMSEA, the Comparative Fit Index (CFI), and the Tucker-Lewis Index (TLI). The following fit index cut-off values are indicative of good model fit: CFI and TLI > 0.90 and RMSEA < 0.05 (35). Pearson’s correlation coefficients were used to examine the relationships between the (sub)scales, of the STA, the EssenCES and Team reflexivity. Also, Pearson’s correlations was used to examine the relationships between the (sub)scales, of the STA and the age and level of experience of participants. An independent two sample *t*-test was used to test whether the scores on relational security differed between male and female participants. The test—retest reliability was tested by calculating the intraclass correlation coefficients (ICC’s) based on a two way mixed-effects model with absolute agreement (36). Analyses were done using IBM SPSS Statistics for Windows, Version 25. For the CFA, (37) computer software was used.

## Results

### Internal consistency

Mean scores, standard deviations and the internal consistencies of the (sub)scales are shown in Table 1. The internal consistency of the STA total scale was relatively high (0.90). The Cronbach’s alphas of the subscales ranged from 0.67 (patient focus) to 0.82 (pro-social team culture).

**TABLE 1:** Properties of the See, Think, Act, EssenCES and Team reflexivity (sub)scales.

	Current α *; M* (SD)	Tighe and Gudjonsson, (5) α *; M* (SD)	Chester et al. (27) CITC	Siu et al. (38) α *; M* (SD)
**STA**				
Therapeutic Management of Risk	0.72; 2.1 (0.3)	0.91; 2.3 (0.6)	0.90	0.93; 2.6 (0.4)
Pro-Social Team Culture	0.82; 2.1 (0.4)	0.93; 2.1 (0.7)	0.96	0.94; 2.6 (0.4)
Boundaries	0.76; 2.4 (0.4)	0.90; 2.3 (0.7)	0.92	0.96; 2.7 (0.3)
Patient Focus	0.67; 2.1 (0.4)	0.87; 2.3 (0.6)	0.92	0.96; 2.6 (0.3)
Total	0.90; 2.2 (0.3)	0.97; 2.2 (0.6)		
**EssenCES**				
Patient Cohesion	0.42; 9.2 (4.5)	0.86; 11.4 (4.2)		
Experienced Safety	0.73; 8.3 (3.7)	0.80; 10.2 (4.4)		
Therapeutic Hold	0.64; 14.8 (2.3)	0.50; 15.9 (2.6)		
**Team Reflexivity**				
Evaluation and learning	0.87; 70.5 (7.4)			
Discussing processes	0.76; 19.3 (3.2)			

### Confirmatory factor analysis

The model results indicated no satisfactory fit for the STA four factor model as suggested by Tighe and Gudjonsson (5). CFI = 0.72, TLI = 0.70, and RMSEA = 0.08. See Table 2 for the item loadings per factor. Most items loaded significantly on their target factors, except items 3 and 26. A revised model leaving these items out did not improve the model fit: CFI = 0.73, TLI = 0.70, and RMSEA = 0.09.

**TABLE 2:** Standardized factor loadings CFA of the See, Think, Act scale.

Item	Risk	Team	Boundaries	Patient
1. We know how to respond if the patient mix needs addressing.	0.58*			
2. We can maintain control by engaging with this patient group.	0.41*			
3. We understand the potential for some visitors to undermine the treatment plans and recovery of patients and take the appropriate action to address this.	0.22			
4. We are vigilant about how visits affect the patient before their visit.	0.56*			
5. We promote tolerance.	0.48*			
6. We look out for patients trying to conceal a deterioration in their mental state.	0.44*			
7. We are vigilant about how visits affect the patient after their visit.	0.70*			
8. We understand the risks some visitors might pose to patients.	0.60*			
9. We are respectful of each other.		0.40*		
10. We deal robustly with discrimination.		0.43*		
11. We set a good example and are positive role models.		0.43*		
12. There is a discipline and pride on our ward reflected in a tidy and well cared for environment.		0.53*		
13. We deal robustly with bullying.		0.50*		
14. We have a ward philosophy that we all understand.		0.80*		
15. We deal robustly with harassment.		0.52*		
16. We have a ward purpose that we all understand.		0.85*		
17. We have ward core values that we all understand.		0.77*		
18. We know which boundaries are non-negotiable and which we can make individual and team judgments about.			0.48*	
19. We understand what maintaining clear boundaries with patients means.			0.66*	
20. We speak up if we think we can see that a colleague has been put in a difficult position that could weaken security.			0.67*	
21. We talk as a team during the shift and at handover.			0.56*	
22. We understand why maintaining a clear boundary with patients is important.			0.72*	
23. We adjust patients care plans according to their risk.				0.63*
24. We know the histories of our patients.				0.48*
25. Care plans are up to date to reflect how our patients are feeling today.				0.49*
26. We monitor how our patients are feeling day to day.				0.33
27. We recognize the relapse factors for each of our patients.				0.41*
28. We engage in reflective practice as team.				0.55*

*Indicates a significant loading on the target factor.

### Construct validity

Construct validity was assessed by means of convergent validity tests. The STA (sub)scales were correlated with the subscales of the EssenCES and the subscales of the Team Reflexivity Scale, results are shown in Table 3. As can be seen from the table, the total score on relational security showed a positive relationship with moderate strength with therapeutic hold (*r* = 0.43) and a weak positive relationship with patient cohesion (*r* = 0.22). The EssenCES subscale therapeutic hold was related to all four STA subscales namely pro-social team culture (*r* = 0.45), therapeutic management of risk (*r* = 0.32), boundaries (*r* = 0.30) and patient focus (*r* = 0.29). The EssenCES subscale patient cohesion was found to be positively related to pro-social team culture (*r* = 0.25), and patient focus (*r* = 0.21). Relational security was also related to the two scales of the Team Reflexivity Scale. To be precise, the STA total score correlated strongly with evaluation and learning (*r* = 0.53) and moderately with discussing processes (*r* = 0.30). Both dimensions of team reflexivity where positively related to all four STA subscales within this sample, with stronger relationships between the STA subscales and evaluation and learning.

**TABLE 3:** Pearson’s correlation coefficients between study variables.

	STA					EssenCES			Team reflexivity	
			
	Risk	Team	Boundaries	Patient	Total	PC	ES	TH	EL	DP
Risk	1									
Team	0.56**	1								
Boundaries	0.57**	0.55**	1							
Patient Focus	0.55**	0.56*	0.50**	1						
Total	0.81**	0.87**	0.78**	0.79**	1					
PC	0.19	0.25*	0.07	0.21	0.22*	1				
ES	–0.12	0.14	–0.00	–0.01	0.03	–0.07	1			
TH	0.32**	0.45**	0.30**	0.29**	0.43**	0.30**	0.05	1		
EL	0.37**	0.55**	0.42**	0.38**	0.53**	0.07	–0.05	0.32**	1	
DP	0.26*	0.23*	0.20*	0.32**	0.30**	0.09	–0.10	–0.04	0.48**	1

PC, Patient Cohesion; ES, Experienced Safety; TH, Therapeutic Hold; EL, Evaluation and Learning; DP, Discussing Processes. *p < 0.05; **p < 0.01.

### Test-retest reliability

A subgroup of 19 participants filled out the STA scale two times to get insight in the test –retest reliability of the scale. The mean interval between the first and the second measurement of the STA scale was 12 days (min. = 7 days; max. = 18 days). ICC’s of the STA total scale and the subscale pro-social team culture indicated good consistency between the ratings over the two time points. However, the ICC’s of the other 3 subscales indicated moderate consistency (see Table 4).

**TABLE 4:** Test-retest reliability of the STA (sub)scales.

	Measure point 1: *M* (SD)	Measure point 2: *M* (SD)	Intraclass correlation coefficient current study (*N* = 19)	Intraclass correlation coefficient (38) (*N* = 30)
**STA**				
Therapeutic Management of Risk	2.2 (0.4)	2.0 (0.3)	0.58	0.50
Pro-Social Team Culture	2.2 (0.4)	1.9 (0.5)	0.80	0.57
Boundaries	2.5 (0.3)	2.3 (0.4)	0.63	0.58
Patient Focus	2.2 (0.3)	2.0 (0.3)	0.66	0.72
Total	2.3 (0.3)	2.1 (0.3)	0.75	0.60

### Further analyses

The independent two sample *t* test revealed no significant difference between male and female participants regarding their view on relational security. A weak positive correlation was found between participants age and the total STA scale (*r* = 0.21, *p* ≤ 0.05) and the STA subscale therapeutic management of risk (*r* = 0.24, *p* = 0.02). No relationship was found between relational security and staff members years of work experience in their current function.

## Discussion

The aim of this present study was to examine the psychometric qualities of the Dutch version of the STA scale. The internal consistency of the STA total scale was good. The internal consistency of the subscales was relatively low compared to other studies using the original English or the Chinese version of the STA scale (5, 27, 38), but was still at broadly acceptable levels. This study was a first attempt in replicating the original factor structure as suggested by Tighe and Gudjonsson (5). Further research is needed into the structural psychometric properties (of the Dutch version) of the STA scale, as the four factor structure suggested by Tighe and Gudjonsson (5) was not confirmed within this current sample. A potential explanation for not finding a satisfactory fit for the STA four factor model could be that items of the STA scale might be related to more than one factor.

With regard to the construct validity of the STA scale, results were promising, as positive relationships between STA total score and related concepts such as elements of ward climate and team reflexivity were found. To be more precise, a moderate positive relationship between the total score of relational security and the ward climate subscale therapeutic hold was found. When looking more closely to the STA subscales, therapeutic hold of the EssenCES was positively related to all four subscales, this result is in line with the results found by Tighe and Gudjonsson (5). Therapeutic hold scale of the EssenCES consist of five items: On this ward, patients can openly talk to staff about all their problems; Staff take a personal interest in the progress of patients; Staff members take a lot of time to deal with patients; Often, staff seem not to care if patients succeed or fail in treatment (reversed scored); Staff know patients and their personal histories very well. The relationship between relational security and therapeutic hold also seems to have face value as these five items reflect some important elements seen in the definitions of relational security such as a therapeutic relationship with trust between staff and patients and a need for in depth knowledge and understanding about patients in order to adjust security and care.

Within our sample only two out of four subscales of the STA scale were related to patient cohesion. These results differ from the results found by Tighe and Gudjonsson (5) as they found all subscales of the STA to correlate positively with patient cohesion. It should be mentioned that the patient cohesion scale of the EssenCES showed weak internal consistency within the current study, therefore the results should be interpreted with care.

The current study adds to previous work on the construct validity of the STA scale by studying the relationship between the scale and team reflectivity, as team reflexivity is regarded an important aspect in enhancing relational security. As expected, team reflexivity was found to be positively correlated with relational security. Both dimensions of team reflexivity where positively related to all four STA subscales within this sample, the strongest relationship was found between evaluation and learning and the STA subscale pro social team culture. The evaluation and learning scale focuses on the evaluation of finished business and learning from previous actions and adaptations. Markham (11) advocates to place more emphasis on reflective practice in the STA guideline and to invest more within forensic metal health settings in explicit guidance regarding evaluation and learning to improve relational practice.

The test-retest reliability for the STA total scale was acceptable. However, the consistency between two assessments, differed between the subscales with moderate consistency for therapeutic management of risk, boundaries and patient focus and acceptable consistency for pro-social team culture. Siu et al., (38) were the first looking into the test-retest reliability of the Chinese version of the STA scale. Their results indicate moderate consistency (ICC ranging from 0.50 till 0.58) for 3 out of 4 subscales, they found acceptable consistency (ICC = 0.72) for the subscale patient focus.

In line with the results of Chester et al., (27) and Siu et al., (38) no relationship was found between relational security and staff members years of work experience in their current function. However, in the current study a weak positive relationship was found between age and management of risk and the total score of the STA scale, this was not reported by other studies yet. Siu et al., (38) found that male participants reported higher levels of perceived confidence in relational security compared to their female colleagues, this result was not replicated within this current sample. There were no differences found between female and male staff members in their relational security scores, this result was in line with the results found in the study of Tighe and Gudjonsson (5) who also found no difference between male and female staff members.

Some limitations of this current study must be noted. Firstly, this study had a relatively small sample size including staff members working in the same organization, limiting generalizability of the results. For examining the factor structure of the STA scale a larger sample would be preferred, the results on the replication of the factor structure therefore need to be seen as preliminary. Secondly, as this study was conducted within a facility for high secure forensic psychiatric care, this study does not give insight in differences in relational security between different security levels. Hence, earlier studies have found that the STA scale is able to differentiate between levels of security (5, 27).

Despite limitations, this current study adds knowledge to earlier studies by measuring relational security with the STA scale in a high secure forensic psychiatric setting, as previous studies have focused on testing the STA scale in facilities providing medium− and low−security care. The study results indicate that further research is needed into the reliability of the (Dutch version of the) STA scale. However, the results on the construct validity of the STA scale were promising, encouraging further development of this instrument designed to measure such an important concept.

It has been argued that in forensic care there is a need to re-focus on relational security in order to improve safety and care processes. Hence, Markham (11) argues that there is a need for robust, comprehensive and consistent implementation of See Think Act in forensic mental health settings in England and Wales. Therefore, the need to have instruments that can measure this concept in a reliable and valid way, remains accentuated. Markham (11), suggests to develop a relational security audit tool on the items of relational security (staff team’s ability to maintain boundaries and deliver therapy, patient mix and dynamics, the internal world of the patient and the unit, and connections to the outside world and the impact of visitors) that can be used to monitor and enhance relational security. The authors of this current study underline the importance of clinical practice and research endeavors aimed at getting a clearer picture of relational security in forensic psychiatric care and how it can be successfully implemented and monitored in daily practice.

The DoH (6) in the United Kingdom referred to relational security as the knowledge and understanding staff have of a patient and of the environment, and the translation of that information into appropriate responses and care. In the Dutch translation of the STA guideline this definition was enriched with approval of the original author: “the knowledge and understanding staff have of a patient, themselves and of the environment, and the translation of that information into appropriate responses and care.” This adjustment was made after experiencing in clinical practice that reflection on oneself as a professional, is important in working with patients and working in a team. The relationship found between relational security and team reflexivity seems to underline the importance of facilitating reflective practice for professionals working in high secure forensic psychiatric care.

We would like to address some points that could be interesting for further development of the STA scale. The first point concerns working toward unambiguity on item level. Hence, in some items it is not clear whether the statement addresses the attitude or behavior of professionals or patients or professionals and patients together. For instance: “There is a discipline and pride on our ward”; “We are respectful of each other”; “We promote tolerance.” There are also items that give difficulties in interpretation, like: “We deal robustly with bullying.” Hence, when respondents answer “not like our team” does that mean “bullying does not occur on our ward” or “we don’t deal with it, we tolerate it” or “we deal with it, but not robustly, but in a ‘gentle’ way.” The developer of the original STA scale has put effort into breaking down statements which addressed more than one subject into separate questions (5). However, there are still items left that seem to tap into more than one aspect of relational security, for instance “We understand the potential for some visitors to undermine the treatment plans and recovery of patients and take the appropriate action to address this.” There are also some items with very specific or overlapping wording that need attention. Hence, 3 out of 5 items concerning management of boundaries explicitly include the word boundary or boundaries. The scale might benefit from adding some alternative wording.

It would be interesting to look at the possibility to encompass the overarching elements of the STA model See, Think and Act into the measure. Relational security as presented in the STA guideline describes the importance of observations, being vigilant, noticing even the smallest changes in behavior or the surrounding (See). The importance of reflection, using insight and knowledge in interpreting or giving meaning to the observations (Think). And the importance of taking appropriate action that fits the situation, to prevent incidents from happening (Act). When looking at the current STA scale some items tap into observations (SEE): We are vigilant about how visits affect the patient after their visit; other into knowledge and reflection (THINK): We know the histories of our patients; we engage in reflective practice; others into action: We adjust patients care plans according to their risk. However, there are also items including more than one element, for instance: We understand the potential for some visitors to undermine the treatment plans and recovery of patients and take the appropriate action to address this. It would be interesting to study the possibilities of developing the instrument in such a way that it could give both insight into the capacities of a team on the content of relational security themes such as boundary management and patient focus, and also insight into the capacities of a team regarding the dynamic process of observing, reflecting and acting. These insights could subsequently give direction to further team development regarding relational security. The items for the original STA Scale came from the statements presented at the end of each section of the STA handbook. Within the handbook these statements are presented as a prompt for professionals to reflect on their practice regarding relational security. Hence, the statements reflect how the service should feel when a team is “getting it right”. Besides revising the statements of the STA scale it could also be worthwhile to revise the statements in the handbook in order to make them as clear as possible, to guide clinical practice.

## Data availability statement

The raw data supporting the conclusions of this article will be made available by the authors, without undue reservation.

## Author contributions

MV, RV, and BB contributed to conception and design of the study. MV gathered the data, organized the database, performed the statistical analysis, and wrote the drafts of the manuscript. All authors contributed to the article and approved the submitted version.
